# Comparison of clinical outcomes of several risk stratification tools in newly diagnosed AML patients: A real‐world evidence in our current therapeutic era

**DOI:** 10.1002/cam4.7103

**Published:** 2024-03-20

**Authors:** Alexandre Iat, Michael Loschi, Sami Benachour, Anne Calleja, Edmond Chiche, Isabelle Sudaka, Danièle Aquaronne, Corinne Ferrero, Laurène Fenwarth, Alice Marceau, Elise Fournier, Berengere Dadone‐Montaudie, Thomas Cluzeau

**Affiliations:** ^1^ Hematology department Nice University Hospital Nice France; ^2^ Mediterranean Center of Molecular Medecine, INSERM Nice France; ^3^ Cote d'Azur University Nice France; ^4^ Hematology Laboratory Nice University Hospital Nice France; ^5^ Hematology Laboratory Lille University Hospital Lille France; ^6^ Molecular Laboratory Nice University Hospital Nice France

**Keywords:** AML, classifier, ELN, prognosis, WHO

## Abstract

**Background of the study:**

AML classification tools have been developed to stratify the risk at AML diagnosis. There is a need to evaluate these tools in the current therapeutic era.

**Cohort characteristics:**

In this retrospective study, we compared five classifiers: ELN 2017, ELN 2022, ALFA classifier, Papaemmanuil et al. classifier, and Lindsley et al. classifier, in a real‐life cohort of 281 patients newly diagnosed for AML in Nice University Hospital. In our cohort median age was 68 years old, sex ratio was M/F 56%/44%, performance status was lower than 2 in 73.1% of patients, AML subtype was “De novo” in 71.5%, “secondary” in 22.4%, and “therapy‐related” in 6.0% of patients. Intensive chemotherapy was used in 53.0% of patients, and non‐intensive chemotherapy in 40.6% of patients. Molecular analysis was available in a large majority of patients and the main mutations found were *NPM1* (22.7%), *DNMT3A* (17.4%), *TP53* (13.1%), *TET2* (12.4%), and *FLT3‐ITD* (12.4%).

**Results:**

In our findings, the comparison of overall survival between the three prognostic groups in the global cohort was statistically significant in all classifiers: ELN 2017 *p* < 0.0001, ELN 2022 *p* < 0.0001, ALFA classifier *p* < 0.0001, Papaemmanuil classifier *p* < 0.0001, Lindsley classifier *p* = 0.001. ELN 2017, ELN 2022, ALFA classifier, Papaemmanuil classifier, and Lindsley classifier were calculated respectively in 99%, 99%, 89%, 90%, and 89% of patients.

**Conclusions:**

Using Akaike’s information criteria (AIC) to compare all five classifiers, ELN 2022 is the best classifier into younger and older patients and for prognosis.

## 1 INTRODUCTION

The prognostic risk stratification for newly diagnosed acute myeloid leukemia (AML) patients is very challenging and is not systematically correlated to same prognosis under all treatments.^1^, ^2^ During the last decades, several AML classifiers have been developed and validated among large cohorts of patients,^3^ including cytogenetics and molecular characteristics, which have a major role in all AML classifiers.^4^ The cohorts of AML patients used to develop these stratification tools were not systematically recent, they did not include systematically elderly patients, and the standard of care in AML evolved since their evaluations. Indeed, in recent years, some treatments have emerged as new standard of care for AML, which include the combination of azacytidine + venetoclax for patients ineligible for intensive chemotherapy.^5^ Some targeted therapies have been validated either in an association with the backbone of 3 + 7 or alone such as midostaurine for *FLT3* mutated AML patients,^2^ ivosidenib for *IDH1* mutated AML patients, enasidenib for *IDH2* AML^6^ mutated patients, and gemtuzumab ozogamycin for CD33‐positive AML patients.^7^ New galenic have been designed like the liposomal form of cytarabine + daunorubicine for therapy‐related and secondary AML.^8^ During the last decades, we also observed a huge improvement for the allogeneic hematopoietic stem cell transplantation (aHSCT).^9^ All these AML classifiers need to be evaluated in our current therapeutic era. The cohort of this study was composed of 281 newly AML diagnosed patients based on WHO 2016^10^ criteria, and the response to the treatments was assessed following ELN 2017 recommandations.^11^ We aim in this study to compare five AML classifiers in a recent real‐life AML cohort.

## 2 METHODS

### 2.1 Study population and procedures

We identified adult patients newly diagnosed with AML from January 2015 to January 2022 in the Nice University Hospital database. We defined AML using WHO 2016 classification. All clinical (age, gender, Performance Status, tumor syndrom, history of previous cancer or hematologic malignancy, treatment received), biological (cell blood count, LDH), cytologic (bone marrow aspiration with percentage of blasts), cytogenetic (karyotype), and molecular parameters (panels of myeloid genes) were collected. Treatment choices were stratified in three categories: intensive chemotherapy, non‐intensive chemotherapy, and best supportive care. Hematopoietic stem cell transplantation (HSCT) was also collected. Intensive chemotherapy was based on “3 + 7” regimens (including CPX‐351) alone or in combination with targeted therapies (such as *IDH1/IDH2* inhibitors, *FLT3* inhibitors, or gemtuzumab ozogamycin). Non‐intensive chemotherapy including HMA alone or in combination, and targeted therapies alone. The risk stratification tools including Papaemmanuil classifier^12^ (https://www.aml‐risk‐model.com/calculator), Lindsley classifier,^13^ ALFA classifier,^14^ ELN 2017,^11^ and ELN 2022^15^ were calculated as previously reported. Written informed consent was provided by all patients before diagnosis. The approval was registered at « Ministère de l'enseignement supérieur et de la recherche » under reference number AC‐2018‐3110.

### 2.2 Data sharing

The data that supports the findings of this study are available on request from the corresponding author.

### 2.3 Statistical analysis

Baseline characteristics were summarized for all AML patients using means, medians, and proportions and compared using analysis of Kruskal‐Wallis test and Pearson's *X* ^2^ tests as appropriate. Responses were evaluated using ELN 2017 criteria. Overall survival (OS) was calculated from the date of AML diagnosis to the date of death or last follow‐up. Survival curves were estimated according to the Kaplan–Meier method and were compared with the log‐rank test. Confidence intervals were computed with 95% coverage. Multivariate Cox proportional hazards models were fitted to evaluate the effect of all classifiers on OS. All classifiers were compared using Akaike's information criteria (AIC) (used to assess the relative goodness fit of the various Cox models, where the lower AIC, the better the fit) and the C‐statistic (a measure indicating overall adequacy of prediction models with censored survival data). Statistical tests were considered significant when the two‐tailed *p*‐value was <0.05. All statistical analyses were performed using SPSS v.26 software (IBM SPSS Statistics).

## 3 RESULTS

### 3.1 Study Cohort

**FIGURE 1:**
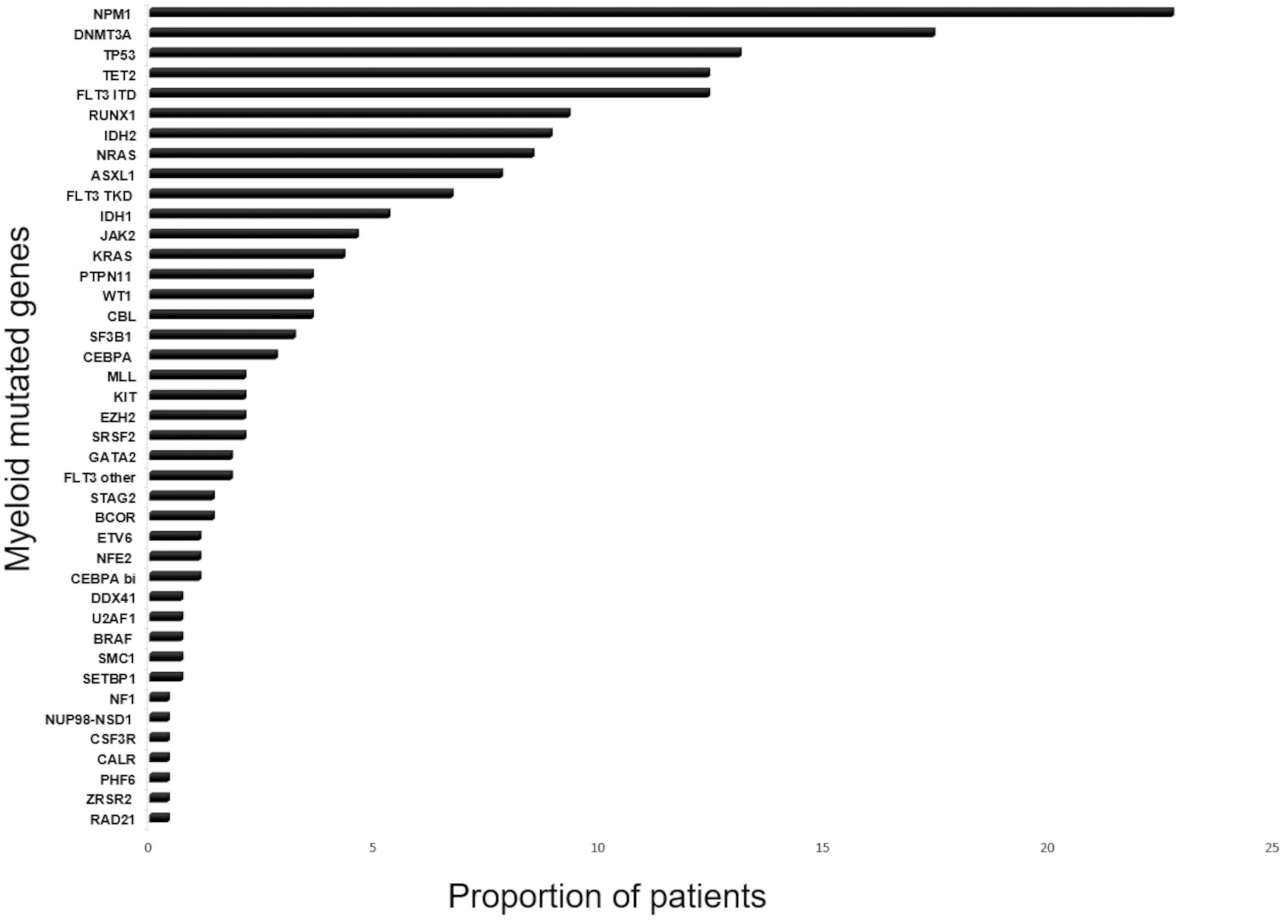
Proportion of mutations identified in the entire cohort.

Two hundred and eighty‐one patients were included from January 2015 to January 2022 at Nice University Hospital. AML cohort was defined following WHO 2016 criteria (Table 1. Global cohort classification following WHO 2016 criteria). At baseline, median age was 68 years old (range, 18–93). Sex ratio M/F was 56%/44%. Seventy percent of patients had a performance status (PS) at 0–1, 11.7% had PS 2, and 7.1% had PS 3–4. Diagnosis of AML was de novo in 200 (71.5%) patients, secondary in 63 (22.4%) patients, and therapy‐related in 17 (6.0%) patients. One hundred fifty (53.4%) patients received an intensive chemotherapy, 114 (40.6%) patients received a non‐intensive chemotherapy, and 17 (6.1%) patients received best supportive care (Table 2. Characteristics of patients in the global cohort). Fifty‐three patients (18.9%) received an HSCT. Molecular analysis was available in a large majority of patients, and the main mutations found were *NPM1* (22.7%), *DNMT3A* (17.4%), *TP53* (13.1%), *TET2* (12.4%), and *FLT3‐ITD* (12.4%) (Figure 1).

**TABLE 1:** Global cohort classification following WHO 2016 criteria.

AML with t(8;21)(q22;q22.1);RUNX1‐RUNX1T1	7/281
AML with inv (16)(p13.1q22) or t(16;16)(p13.1;q22);CBFB‐MYH11	9/281
APL with PML‐RARA	0/281
AML with t(9;11)(p21.3;q23.3);MLLT3‐KMT2A	2/281
AML with t(6;9)(p23;q34.1);DEK‐NUP214	2/281
AML with inv (3)(q21.3q26.2) or t(3;3)(q21.3;q26.2); GATA2, MECOM	5/281
AML (megakaryoblastic) with t(1;22)(p13.3;q13.3);RBM15‐MKL1	0/281
Provisional entity: AML with BCR‐ABL1	4/281
AML with mutated NPM1	64/281
AML with biallelic mutations of CEBPA	3/281
Provisional entity: AML with mutated RUNX1	26/281
AML with myelodysplasia‐related changes	89/281
Therapy‐related myeloid neoplasms	14/281
AML, NOS	56/281
Myeloid sarcoma	0/281
Myeloid proliferations related to Down syndrome	0/281

**TABLE 2:** Characteristics of patients in the global cohort.

Variable	Value
Median age (range)	68 yo (18–93)
Male/Female	159/122
Performance status	0–1: 78.8% 2: 11.7% 3–4: 7.1%
AML subtype	De novo: 201 (71.5%) Secondary: 63 (22.4%) Post chemotherapy: 17 (6.0%)
Therapeutical strategies	Intensive chemotherapy: 150 (53.4%) Non intensive chemotherapy: 114 (40.6%) Best supportive care: 17 (6.1%)

### 3.2 Overall survival

**FIGURE 2:**
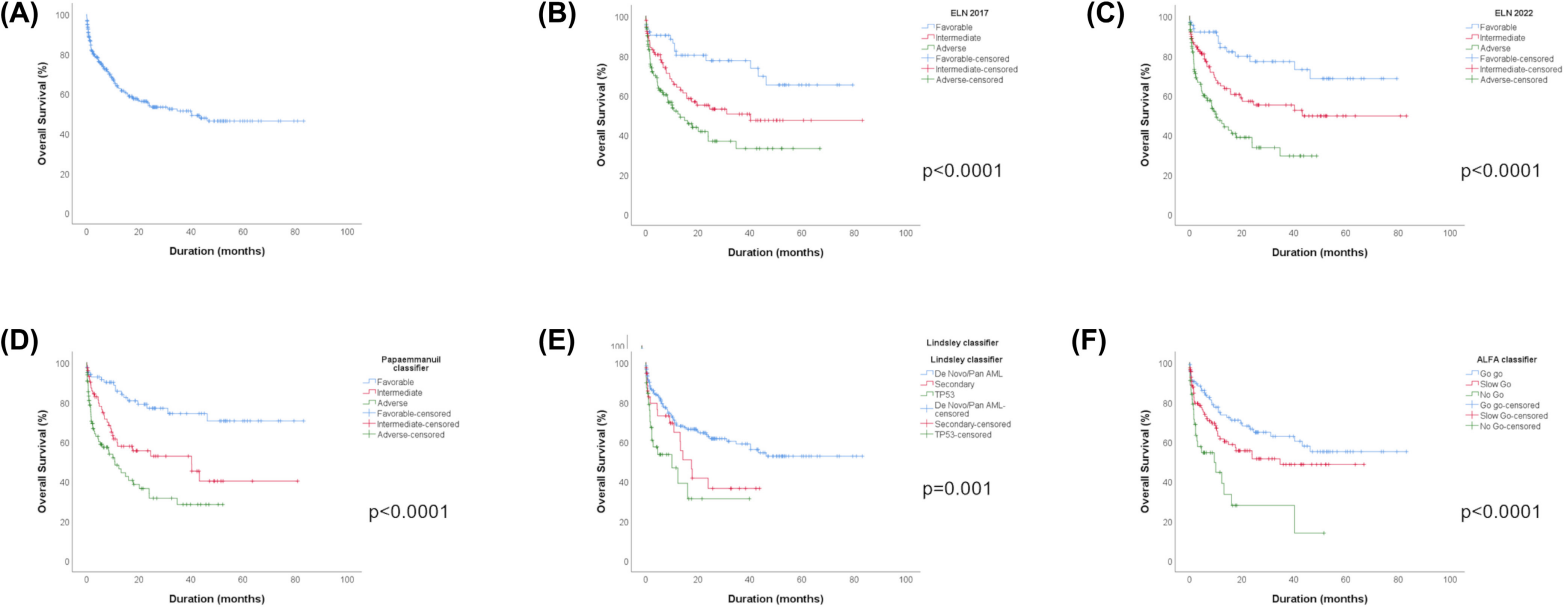
Overall survival of the entire cohort stratified into risk groups for each classifier. (A) OS of the global cohort, (B) OS depending to ELN 2017, (C) OS depending to ELN 2022, (D) OS depending to Papaemmanuil classifier, (E) OS depending to Lindsley classifier. (F) OS depending to ALFA classifier.

Median OS was 40.31 months in the entire cohort. Median OS has not been reached in patients treated by intensive chemotherapy; in patients treated by non‐intensive chemotherapy, the median OS was 20.21 months and 0.95 months in patients treated by best supportive care. The stratification of the entire cohort with each classifier has been evaluated, and the median OS is represented on Figure 2.

### 3.3 Survival probabilities depending to the prognostic tools

In ELN 2017 classifier, the median OS of favorable group is not reached, 40.3 months in the intermediate group and 13.2 months in the adverse group. In ELN 2022 classifier, the median OS of favorable group is not reached, 43.2 months for the intermediate group and 10.2 months in the adverse group (Table 3). The comparison of overall survival between the three prognostic groups was statistically significant in all classifiers: ELN 2017 *p* < 0.0001, ELN 2022 *p* < 0.0001, ALFA classifier *p* < 0.0001, Papaemmanuil classifier *p* < 0.0001, Lindsley classifier *p* = 0.001 (Figure 2). ELN 2017, ELN 2022, ALFA classifier, Papaemmanuil classifier, and Lindsley classifier were calculated respectively in 99%, 99%, 89%, 90%, and 89% of patients.

**TABLE 3:** Median OS in global cohort.

Classifiers	Favorable	Intermediate	Adverse	*p*‐Value
ELN 2017 median OS	Not reached	40.3 months	13.2 months	<0.0001
ELN 2022 median OS	Not reached	43.2 months	10.2 months	<0.0001
Papaemmanuil classifier median OS	Not reached	40.3 months	10.8 months	<0.0001

Classifier	Go‐go	Slow‐go	No‐go	*p*‐Value
ALFA classifier median OS	Not reached	34.7 months	9.5 months	<0.0001

Classifier	De novo/Pan AML	Secondary AML	TP53 mutated	*p*‐Value
Lindsley classifier	Not reached	17.6 months	10.1 months	=0.001

### 3.4 Survival probabilities depending on the age and/or treatment choices

For the patients who received an intensive chemotherapy, median OS was not reached. The overall survival for patients receiving an intensive chemotherapy stratified into each classifier is presented in Table 4.

**TABLE 4:** Median OS in intensive chemotherapy cohort.

Classifiers	Favorable	Intermediate	Adverse	*p*‐value
ELN 2017 median OS	Not reached	Not reached	14.9 months	=0.003
ELN 2022 median OS	Not reached	Not reached	10.8 months	<0.0001
Papaemmanuil Classifier median OS	Not reached	40.3 months	14.9 months	<0.0001

Classifier	Go‐go	Slow‐go	No‐go	*p*‐Value
ALFA classifier median OS	Not reached	Not reached	9.5 months	=0.01

Classifier	De novo/Pan AML	Secondary AML	TP53 mutated	*p*‐value
Lindsley classifier	Not reached	Not reached	4.6 months	=0.014

**FIGURE 3:**
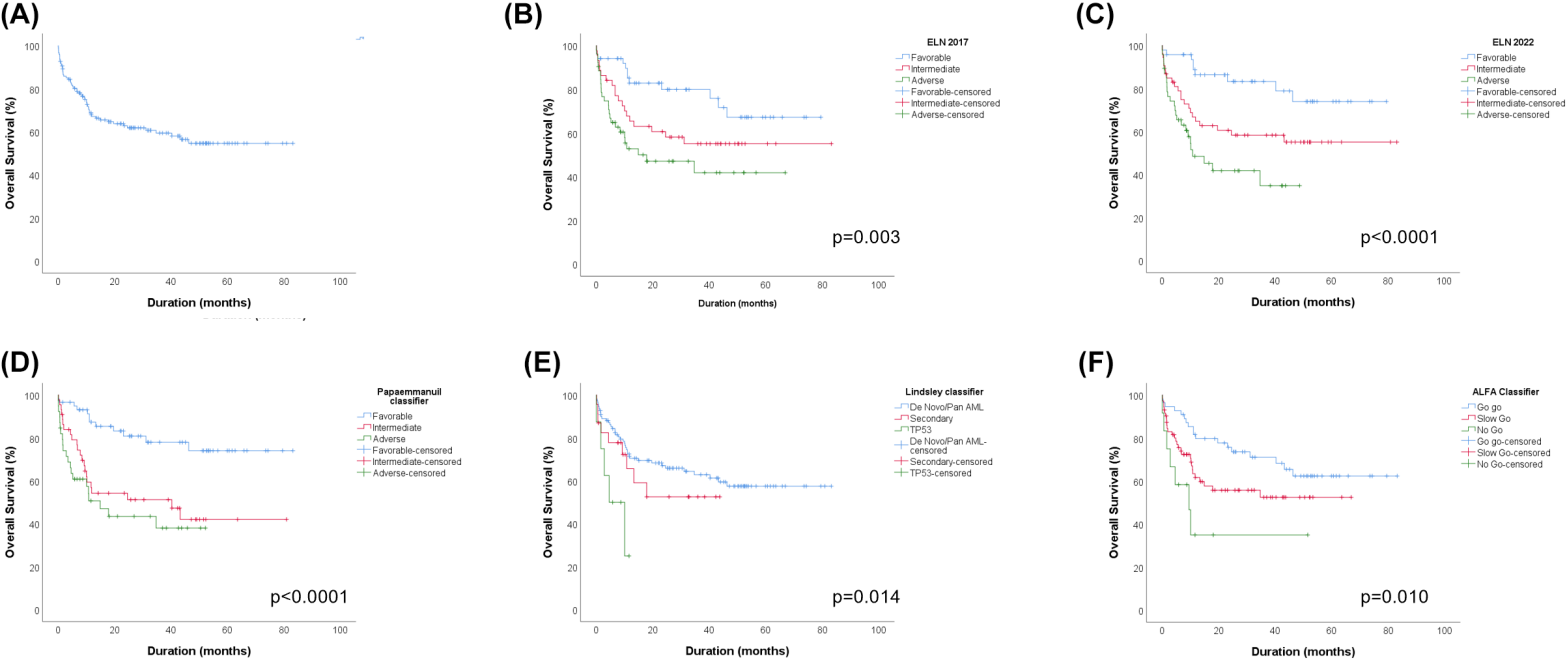
Overall survival of AML patients treated by intensive chemotherapy stratified into each group by each classifier.(A) OS of the global cohort, (B) OS depending to ELN 2017, (C) OS depending to ELN 2022, (D) OS depending to Papaemmanuil classifier, (E) OS depending to Lindsley classifier, (F) OS depending to ALFA classifier.

For each classifier, OS comparison between subgroups was statistically significant, showing a great power of discrimination for patients treated by intensive chemotherapy in all classifiers (Figure 3).

For the patients receiving a non‐intensive chemotherapy, median OS was 20.2 months. The results on OS of the stratification of patients into the five classifiers are represented in Table 5.

**TABLE 5:** Median OS in non‐intensive chemotherapy cohort.

Classifiers	Favorable	Intermediate	Adverse	*p*‐Value
ELN 2017 median OS	Not reached	17.6 months	20.2 months	=0.629
ELN 2022 median OS	17.6 months	20.2 months	13.2 months	=0.242
Papaemmanuil Classifier median OS	Not reached	40.3 months	8.5 months	=0.032

Classifier	Go‐go	Slow‐go	No‐go	*p*‐value
ALFA classifier median OS	Not reached	24.0 months	13.2 months	=0.144

Classifier	De novo/Pan AML	Secondary AML	TP53 mutated	*p*‐value
Lindsley classifier	40.3 months	14.3 months	Not reached	=0.209

The comparison between the stratification group survival curves in each classifier is not statistically significant, except for the Papaemmanuil classifier. (Figure S1).

**FIGURE 4:**
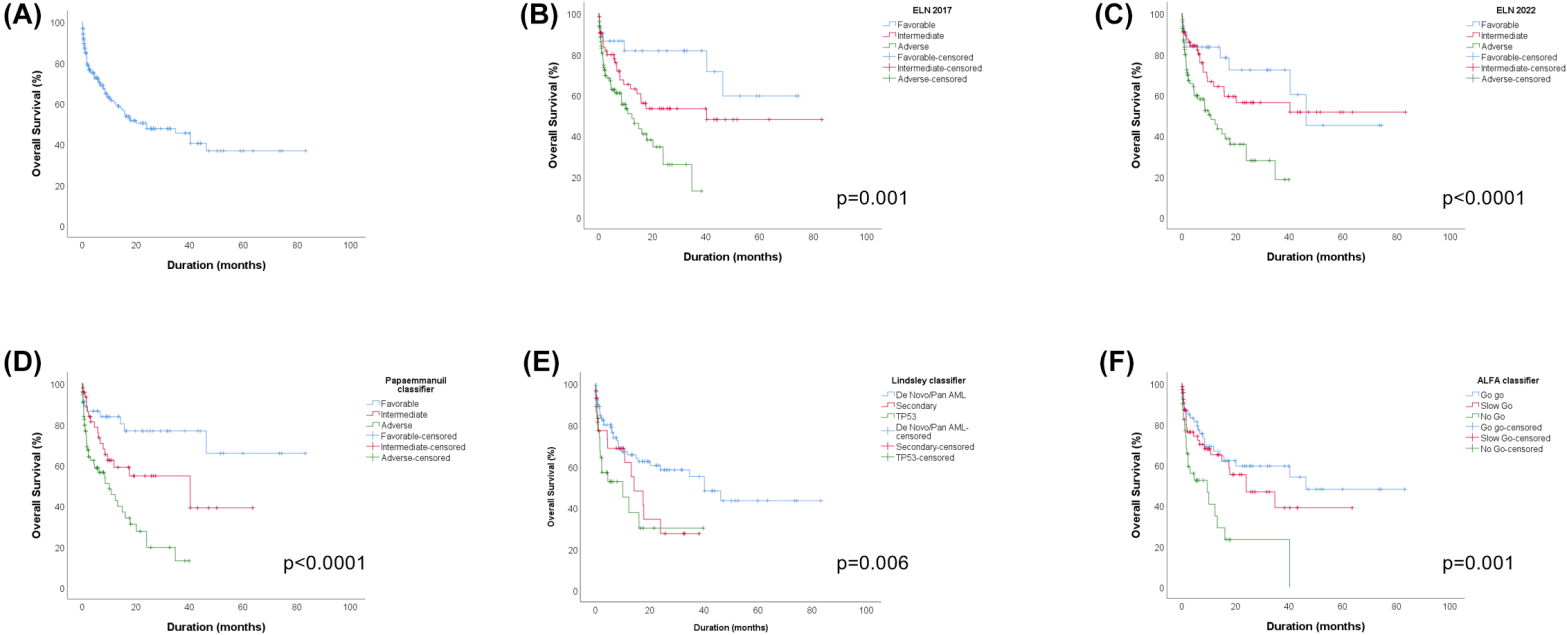
Overall survival of AML patients aged ≥60 years stratified into each group by each classifier. (A) OS of the global cohort, (B) OS depending to ELN 2017, (C) OS depending to ELN 2022, (D) OS depending to Papaemmanuil classifier, (E) OS depending to Lindsley classifier, (F) OS depending to ALFA classifier.

For the patients ≥60 years old, median OS was 38.7 months. The median OS in this category of patients stratified into each classifier is shown in Table 6 (Median OS in 60‐year‐old or older cohort). The comparison between the stratification group survival curves in each classifier is statistically significant (Figure 4).

**TABLE 6:** Median OS in 60 years old or older cohort.

Classifiers	Favorable	Intermediate	Adverse	*p*‐value
ELN 2017 median OS	Not reached	40.3 months	12.4 months	=0.001
ELN 2022 median OS	46.3 months	Not reached	10.8 months	<0.0001
Papaemmanuil Classifier median OS	Not reached	40.3 months	10.1 months	<0.0001

Classifier	Go‐go	Slow‐go	No‐go	*p*‐Value
ALFA classifier median OS	46.3 months	24.0 months	9.5 months	=0.001

Classifier	De novo/Pan AML	Secondary AML	TP53 mutated	*p*‐Value
Lindsley classifier	40.3 months	14.3 months	10.1 months	=0.006

### 3.5 Comparison between survival prediction power of the prognostic tools

The AICcs were used to compare the univariable Cox models containing prognostics scoring systems as covariates (lower is better). The AICcs for the OS models were 2609 (ELN 2022), 2669 (Papaemmanuil classifier), 2732 (ALFA classifier), 3025 (ELN 2017), and 18,854 (Lindsley classifier) (Table 7) in the entire cohort and in aged more or less than 60 years old and based on type of treatment (intensive and non‐intensive chemotherapy). To assess if the relative prognostic discrimination for the five prognostic tools was influenced by type of treatment (intensive vs. non‐intensive chemotherapy) or age (< 60 vs. ≥60 years old), we calculated the AIC scores for the five prognostic tools in each of these four subgroups (Table 7). The ELN 2022 appeared to have the best relative prognostic discrimination (lowest AIC) in patients who have received either intensive and non‐intensive chemotherapy and in patients aged less and more than 60 years old.

**TABLE 7:** Calculated AICc scores (from OS models) for the different prognostic tools (lower is better) in the entire cohort and in aged more or less than 60 years old and based on type of treatment (intensive and non‐intensive chemotherapy).

Entire cohort				
ELN 2022	Papaemmanuil classifier	ALFA classifier	ELN 2017	Lindsley classifier
2609	2669	2732	3025	18,854
Aged ≥ 60 years old				
ELN 2022	Papaemmanuil classifier	ELN 2017	ALFA classifier	Lindsley classifier
1692	1731	1808	2132	2376
Aged < 60 years old				
Lindsley classifier	ELN 2022	ALFA classifier	ELN 2017	Papaemmanuil classifier
892	896	927	967	1010
Intensive chemotherapy				
ELN 2022	ELN 2017	ALFA classifier	Papaemmanuil classifier	Lindsley classifier
1549	1577	1608	1634	2189
Non‐intensive chemotherapy				
Papaemmanuil classifier	Lindsley classifier	ELN 2017	ALFA classifier	ELN 2022
1167	1234	1996	4168	5620

## 4 DISCUSSION

Our study evaluates the real‐life application of classifiers into AML disease. The cohort included 281 patients newly diagnosed AML from the Nice University Hospital. Five classifiers were calculated for the entire cohort of patients and not only for the patients in which the classifications have been developed (intensive/non‐intensive chemotherapy and ageless and more than 60 years old). We divided each classifier into different subgroups of patients to identify the discrimination power between them. The aim was to choose the most appropriate classifier in patients newly diagnosed AML in our current therapeutical strategies era.

We showed here that ELN 2022 is the best classifier into younger and older patients and for the patients receiving an intensive chemotherapy. ELN 2022 is the best classifier of the entire cohort. We also identified that each classifier had a high power of discrimination between the subgroups for prognosis.

The strengths of our study are the large number of patients newly diagnosed AML and treated following the standard of care from 2015 to 2022. The entire cohort was analyzed into each classifier and not only in the population allowing to design the classifiers. Nevertheless, our study carries some limits. It is a monocentric and a retrospective study, and because some molecular data were missing, we have not been able to stratify the whole cohort into each classifier.

After the end of inclusion, two new AML classifications were published: WHO 2022^16^ and ICC 2022.^17^ Despite these new classifications, WHO 2016 classification has been chosen in this study to allow a consensus on AML diagnosis between the three classifications WHO 2016, WHO 2022 and ICC 2022. Moreover, the new classifications WHO 2022, and ICC 2022 consider AML diagnosis for patients who were not diagnosed for AML at the time of the study but for a MDS and were treated depending on the MDS risk stratification following IPSS or IPSS‐R scores.

New classifications would change the AML population, and this may have an impact on the stratification risk.

The ALFA classifier has been developed for elderly patients receiving an intensive chemotherapy.^14^ The characteristics of our population were different. A large proportion of patients were not receiving intensive chemotherapy and were treated by targeted therapy or a non‐intensive chemotherapy. The Lindsley classifier focused on the molecular abnormalities in AML^13^ and was also prognostic in our cohort. This result confirmed the important role of mutations in myeloid genes in the chemoresistance. The Lindsley classifier is powerful for patients treated by non‐intensive chemotherapy but less for patients receiving intensive chemotherapy.

Our analysis of the prognostic discrimination of each classifier in patients less than 60 years old showed less power of discrimination. It could be explained by the impact of aHSCT, which is often performed in this subgroup, decreasing the negative prognostic impact of poor cytogenetic and molecular categories on the outcome. Moreover, characteristic of aHSCT were improved recently^9^, ^18^ with more donor available (Haplo‐identical) and improvement conditioning,^19^ antiviral prophylaxis,^20^ GVHD treatments^21^ and maintenance treatment post‐aHSCT.^22^, ^23^

These results showed that evaluation of mutational landscape in AML disease is necessary to define the prognosis of AML disease and choose the most appropriate treatment. Among the classifiers developed in AML, ELN 2022 was the best classifier for the evaluation of AML disease prognosis independently of the age of patients and of the chemotherapy regimen received. This result should be confirmed in a larger, multicentric cohort of AML patients.

## 5 CONCLUSION

Among all AML prognostic classifiers evaluated in our study, ELN 2022 was the most accurate to evaluate prognosis of AML independently of the age and the intensity of chemotherapy received. With the big improvement of therapeutical strategies in AML, our study identified that classifiers need to be validated in recent cohorts of patients receiving all new approved treatments and can't be extrapolated to all patients outside of the characteristics of the initial population having been allowed to build the classifier.

## AUTHOR CONTRIBUTIONS

**Alexandre Iat:** Data curation (equal); writing – original draft (lead). **Michael Loschi:** Data curation (equal); writing – review and editing (equal). **Sami Benachour:** Data curation (equal); writing – review and editing (equal). **Anne Calleja:** Data curation (equal); writing – review and editing (equal). **Edmond Chiche:** Data curation (equal); writing – review and editing (equal). **Isabelle Sudaka:** Data curation (equal); writing – review and editing (equal). **Danièle Aquaronne:** Data curation (equal); writing – review and editing (equal). **Corinne Ferrero:** Data curation (equal); writing – review and editing (equal). **Laurène Fenwarth:** Data curation (equal); writing – review and editing (equal). **Alice Marceau:** Data curation (equal); writing – review and editing (equal). **Elise Fournier:** Data curation (equal); writing – review and editing (equal). **Berengere Dadone‐Montaudie:** Data curation (equal); writing – review and editing (equal). **Thomas Cluzeau:** Conceptualization (lead); data curation (equal); formal analysis (lead); methodology (lead); validation (lead); visualization (lead); writing – original draft (lead); writing – review and editing (equal).

## CONFLICT OF INTEREST STATEMENT

The authors declare no relevant conflict of interest for this publication.

## Supporting information


Figure S1.

## Data Availability

The data that support the findings of this study are available on request from thecorresponding author (cluzeau.t@chu‐nice.fr).
