# Supplementary figures and images for: Comparison of clinical outcomes of several risk stratification tools in newly diagnosed AML patients: A real‐world evidence in our current therapeutic era
Source: Cancer Med. 2024 Mar 20;13(6):e7103. doi: 10.1002/cam4.7103 (PMC10952023; doi:10.1002/cam4.7103)

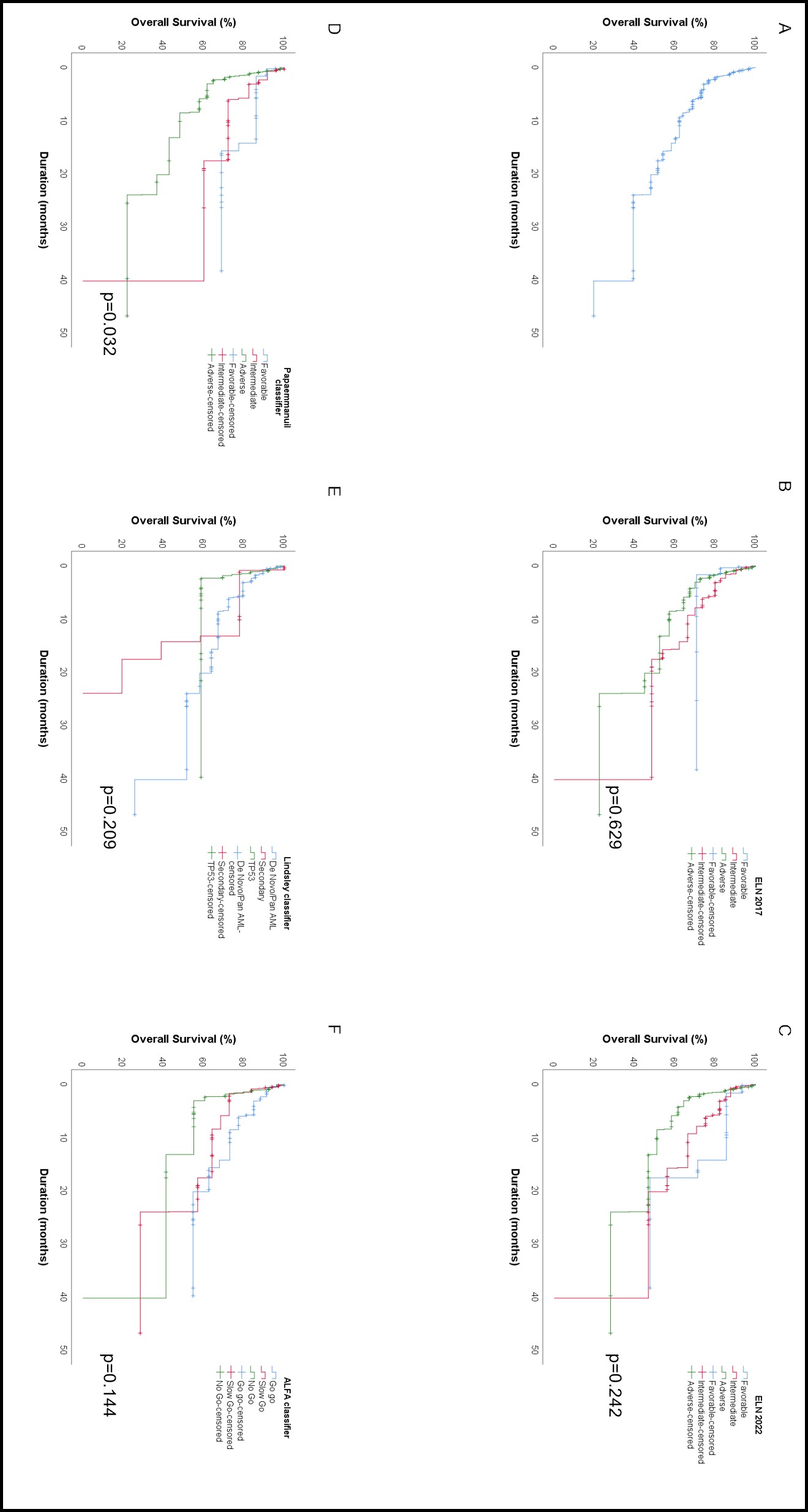

Supplement: Supplementary file 1 — Figure S1. [file CAM4-13-e7103-s001.jpeg]
